# Moderate Exercise Attenuates Lipopolysaccharide-Induced Inflammation and Associated Maternal and Fetal Morbidities in Pregnant Rats

**DOI:** 10.1371/journal.pone.0154405

**Published:** 2016-04-28

**Authors:** Karina T. Kasawara, Tiziana Cotechini, Shannyn K. Macdonald-Goodfellow, Fernanda G. Surita, João L. Pinto e Silva, Chandrakant Tayade, Maha Othman, Terence R. S. Ozolinš, Charles H. Graham

**Affiliations:** 1 Department of Biomedical and Molecular Sciences, Queen’s University, Kingston, ON, Canada; 2 Department of Obstetrics and Gynaecology, University of Campinas, Campinas, SP, Brazil; Virgen Macarena University Hospital, School of Medicine, University of Seville, SPAIN

## Abstract

Fetal growth restriction (FGR) and coagulopathies are often associated with aberrant maternal inflammation. Moderate-intensity exercise during pregnancy has been shown to increase utero-placental blood flow and to enhance fetal nutrition as well as fetal and placental growth. Furthermore, exercise is known to reduce inflammation. To evaluate the effect of moderate-intensity exercise on inflammation associated with the development of maternal coagulopathies and FGR, Wistar rats were subjected to an exercise regime before and during pregnancy. To model inflammation-induced FGR, pregnant rats were administered daily intraperitoneal injections of *E*. *coli* lipopolysaccharide (LPS) on gestational days (GD) 13.5–16.5 and sacrificed at GD 17.5. Control rats were injected with saline. Maternal hemostasis was assessed by thromboelastography. Moderate-intensity exercise prevented LPS-mediated increases in white blood cell counts measured on GD 17.5 and improved maternal hemostasis profiles. Importantly, our data reveal that exercise prevented LPS-induced FGR. Moderate-intensity exercise initiated before and maintained during pregnancy may decrease the severity of maternal and perinatal complications associated with abnormal maternal inflammation.

## Introduction

Fetal growth restriction (FGR) affects 5–10% of clinically recognized pregnancies [1–3] and is often associated with aberrant maternal inflammation. Although normal pregnancy is considered to be a state of low-grade inflammation [4, 5], there is strong evidence that adverse pregnancy complications, including FGR and pre-eclampsia (PE), are linked to aberrant maternal inflammation [6, 7]. Women affected by FGR/PE have a heightened inflammatory state characterised by increased levels of pro-inflammatory cytokines and chemokines such as tumour necrosis factor alpha (TNF), interleukin 6, and the macrophage chemoattractant chemokine ligand 2 (CCL2), systemically and locally in the placenta [8–12]. This abnormal inflammatory response often leads to oxidative and nitrosative stress [10, 13, 14].

Normal pregnancy is associated with a shift in maternal hemostasis towards a pro-thrombotic state [15, 16], and pregnancy-associated coagulopathies have been implicated in the pathophysiology of complications including PE, fetal loss and FGR [17–20]. While it is unclear whether disruptions in maternal hemostasis are causally linked to the deficient utero-placental perfusion that characterises these pregnancy disorders, there is recent evidence that antenatal anti-thrombotic therapy reduces the risk of poor pregnancy outcomes, including FGR, in women at risk of placental dysfunction [21]. Moreover, thrombophilia is commonly associated with fetal demise when utero-placental insufficiency is present [22].

There is substantial cross talk between inflammatory and hemostasis pathways, and dysregulation of both systems is implicated in the pathophysiology of pregnancy complications [20]. Previous work from our laboratory revealed that inflammation-induced coagulopathies in a rat model are associated with altered utero-placental hemodynamics and fetal death [18, 19, 23]. Importantly, our data revealed that inhibition of TNF successfully prevented inflammation-induced hemostatic alterations, normalized utero-placental perfusion and prevented fetal loss [18, 23].

Exercise has immunomodulatory effects. Specifically, exercise reduces the release of pro-inflammatory cytokines, promotes the release of anti-inflammatory cytokines, decreases visceral fat mass and reduces the expression of Toll-Like Receptor 4 (TLR-4) on monocytes and macrophages [24, 25]. The latter is a cell surface protein that plays a fundamental role in the release of pro-inflammatory cytokines and activation of innate immunity. Moderate exercise also regulates hemostasis by decreasing platelet reactivity and fibrin formation [26, 27].

There is evidence revealing the benefits of exercise on achieving optimal vascular function [28]. In particular, moderate-intensity exercise has been shown to promote beneficial vascular adaptations and reduce cardiovascular risks [29]. Additionally, moderate-intensity exercise during pregnancy increases utero-placental blood flow, enhances fetal nutrition and improves fetal and placental growth [30, 31]. In the present study we tested the hypothesis that exercise attenuates fetal and maternal morbidities associated with aberrant inflammation. To the best of our knowledge, this is the first study revealing a beneficial effect of an exercise protocol initiated before and during pregnancy in the attenuation of maternal inflammation, systemic coagulopathies and FGR.

## Materials and Methods

### Animals

All procedures for animal experimentation were undertaken in accordance with the principles and guidelines of the Canadian Council on Animal Care and were approved by the Queen's University Animal Care Committee (Permit Number: 2010-012-R3-A8). Virgin female Wistar rats (3–4 months old; Charles River Laboratories, St-Constant, QC, Canada) were housed in a light- and humidity-controlled facility with free access to food and water. All animals were evaluated daily in order to ensure health and well-being.

### Exercise Protocol

Exercise under regulated conditions (*i*.*e*. constant time and intensity) was achieved using a rodent treadmill (LE 8700 series, Panlab, Harvard Apparatus, Barcelona, Spain). Belt speed was adjusted and individualized according to the maximal exercise ability (MEA; described below) determined for each rat during a training phase.

### Evaluation of Maximal Exercise Ability

Evaluation of maximal exercise ability (MEA) for each rat was performed as previously published by Jiao *et al*. [32]. Briefly, belt inclination was set at 0^◦^ and belt speed was set to 30 cm/sec. Every 30 sec, belt speed was increased 2 cm/sec until rats were unable to run regularly, or until they rested on the shock grid (delivering a shock of <1mA) more than three times. The final belt speed at which each animal was able to run was set as the MEA for that subject. This MEA assessment was repeated three times for each rat (with one-hour rest times between trials) and the average MEA over the three trials was calculated. Mean MEA was calculated both before and after pre-pregnancy exercise training (described below) in order to evaluate the change in each rat’s aerobic capacity prior to mating.

### Pre-Pregnancy Exercise Protocol

The protocol for exercise training was based on the MEA for each rat and was established from an exercise training protocol for pregnant rats published by Amorim, *et al*. [33] and modified by us. Briefly, all rats were subjected to four weeks of pre-pregnancy exercise training. Each training week consisted of five consecutive days of training followed by two days of rest. The daily pre-pregnancy exercise program was divided in three stages consisting of 1) warm-up; 2) training and; 3) cool-down. During the warm-up stage (lasting five minutes), belt speed intensity was set to 40% of the subject’s MEA. During the training phase, belt intensity and duration of exercise were modulated based upon the week of training. Specifically, rats ran at 40% of their MEA for 20 min during week one, 50 min during week two, 60 min during week three and 60 min at 65% of their MEA during week four. During the cool-down period (five minutes), belt speed was slowly decreased 2–3 cm/sec every 30 sec until the treadmill was stopped.

### Mating

Following four weeks of pre-pregnancy exercise training, virgin female rats were co-housed overnight with a male rat (at a 2:1 ratio). The detection of sperm in the vaginal lavage the following morning represented gestational day (GD) 0.5. Pregnant rats were then subjected to the pregnancy exercise protocol (described below) the same day.

### Pregnancy Exercise Protocol

Throughout pregnancy, rats were subjected to a moderate-intensity exercise program. Pregnant rats ran at 65% of their MEA for various durations over gestation (cycle one: 50 min/day; cycle two: 30 min/day; and cycle three: 20 min/day). Each cycle consisted of five consecutive days of exercise followed by one day of rest until study endpoint on GD 17.5.

### Complete Blood Cell Count Analysis

Complete blood cell (CBC) count analysis was performed on maternal whole blood collected on GD 17.5 at the time of euthanasia. Briefly, maternal blood was taken via cardiac puncture using a syringe pre-filled with EDTA. Analysis was performed on each sample (12 μl) using an ABC Vet Animal Blood Counter (Scil Animal Care Company, Gurnee, IL, USA) according to the manufacturer’s instructions. Populations of red blood cells, white blood cells, lymphocytes, monocytes and granulocytes were assessed.

### Inflammation-Induced Rat Model of FGR

We used our previously established model of LPS-induced FGR [7]. Pregnant Wistar rats received daily intraperitoneal (i.p.) injections of low-dose lipopolysaccharide (LPS; 10 μg/kg on GD 13.5 followed by 40 μg/kg on GD 14.5, 15.5 and 16.5) or saline (0.1 ml/100 g) during the second half of gestation, and were euthanized on GD 17.5. To assess whether exercise attenuates LPS-induced inflammation and associated maternal and fetal morbidities, we utilized results from previously published work in which rats were treated with saline (Se + saline) or LPS (Se + LPS) as sedentary control data [7]. In this previous study, FGR was defined as fetal weight falling below the 10^th^ percentile for gestational age. Specifically, the threshold of FGR was determined by evaluating the distribution of all fetal weights from the saline-treated control cohort (n = 22 dams; n = 305 fetuses; mean fetal weight = 0.9244 ± 0.007 g) and fetuses with weights below 0.8071 g (lower 10th percentile) were designated as FGR. Treatment of sedentary rats with LPS in our previous study resulted in a mean fetal weight = 0.8421 ± 0.006 g (n = 28 dams; n = 258 fetuses) [7].

To evaluate the effect of exercise on LPS-induced FGR, exercised rats (as described in the above sections) received daily i.p. injections of saline or LPS (*Escherichia coli* serotype 0111:B4; Sigma-Aldrich, Oakville, ON, Canada) according to the same protocol established for the sedentary group during pregnancy [7]. Fetal weights were measured on GD 17.5 and were normalized to litter size (fetal weight/number of fetuses in litter) to account for alterations in fetal weight attributable to variations in litter size as has been previously described [34].

### Thromboelastography (TEG)

Prior to euthanasia on GD 17.5, pregnant rats were anaesthetized using 40–50 mg/kg sodium pentobarbital (CEVA Santé Animale, Rutherford, NJ, USA). Maternal blood was collected via cardiac puncture, using a 26’-gauge needle, and placed into a tube pre-filled with trisodium citrate. Thromboelastography (TEG) was performed on citrated blood as previously described [18, 19, 23] using a TEG^®^ 5000 Haemostasis System and TEG^®^ Haemostasis Analyzer software Version 4.2 (Haemoscope Corporation, Skokie, IL, USA). Prior to each analysis, an electronic quality test was performed on the TEG^®^ 5000 Haemostasis System by a trained operator. Blood (340 μl) was re-calcified by adding 20 μl of 0.2 M calcium chloride and loaded into a disposable plastic cuvette for analysis. Data were collected for 75–90 min and the following parameters were evaluated: time to clot formation (R), speed of clot propagation (α), rate of clot formation (K), strength/stability of clot (MA), clotting index (CI; a value that is based on the four parameters above,) and LY30 (percent clot dissolution in 30 min). Our previously published data collected from the sedentary cohort of saline- and LPS-treated rats (n = 9 and 13, respectively) [19] were used as the reference range and controls. Exercised rats from the current study were considered to exhibit hemostatic alterations if two or more parameters fell beyond reference ranges previously established during normal pregnancy in saline-treated, sedentary rats.

### Statistical Analysis

All statistical analyses were performed using GraphPad Prism 6.0 Software (GraphPad Software Inc., La Jolla, CA, USA). Data are presented as mean ± standard error of the mean (SEM). Student’s t-test was used to compare means between two groups. To evaluate whether exercise ameliorates LPS-induced outcomes, data were analysed using two-way ANOVA and the Bonferroni correction was applied to all row and column comparisons to determine significant differences between comparison groups. Effect size was measured by Eta Squared and appear in the text as η^2^_x_ where x refers to the main effect being tested. Differences between groups were considered significant when p < 0.05.

## Results

### Exercise Training Increased the Aerobic Capacity of Rats Prior to Pregnancy

All trained rats (n = 10) increased their aerobic capacity following four weeks of pre-pregnancy exercise. Specifically, MEA significantly increased from 63.7 cm/sec ± 8.6 cm/sec prior to training, to 79.1 cm/sec ± 6.4 cm/sec following training (p < 0.001). Since all rats achieved a similar final belt speed following the training phase, animals were randomly assigned to the saline (Ex+Saline; n = 5) or LPS (Ex+LPS; n = 5) group. Five rats were unable to start the pre-pregnancy training phase due to persistent refusal to run on the treadmill. These rats did not exhibit any obvious physical condition suggestive of inability to run. An additional rat was diagnosed with hip displacement. These six rats were excluded from the study.

### Exercise Prevented LPS-Induced Increases in White Blood Cell Counts

**Fig 1:**
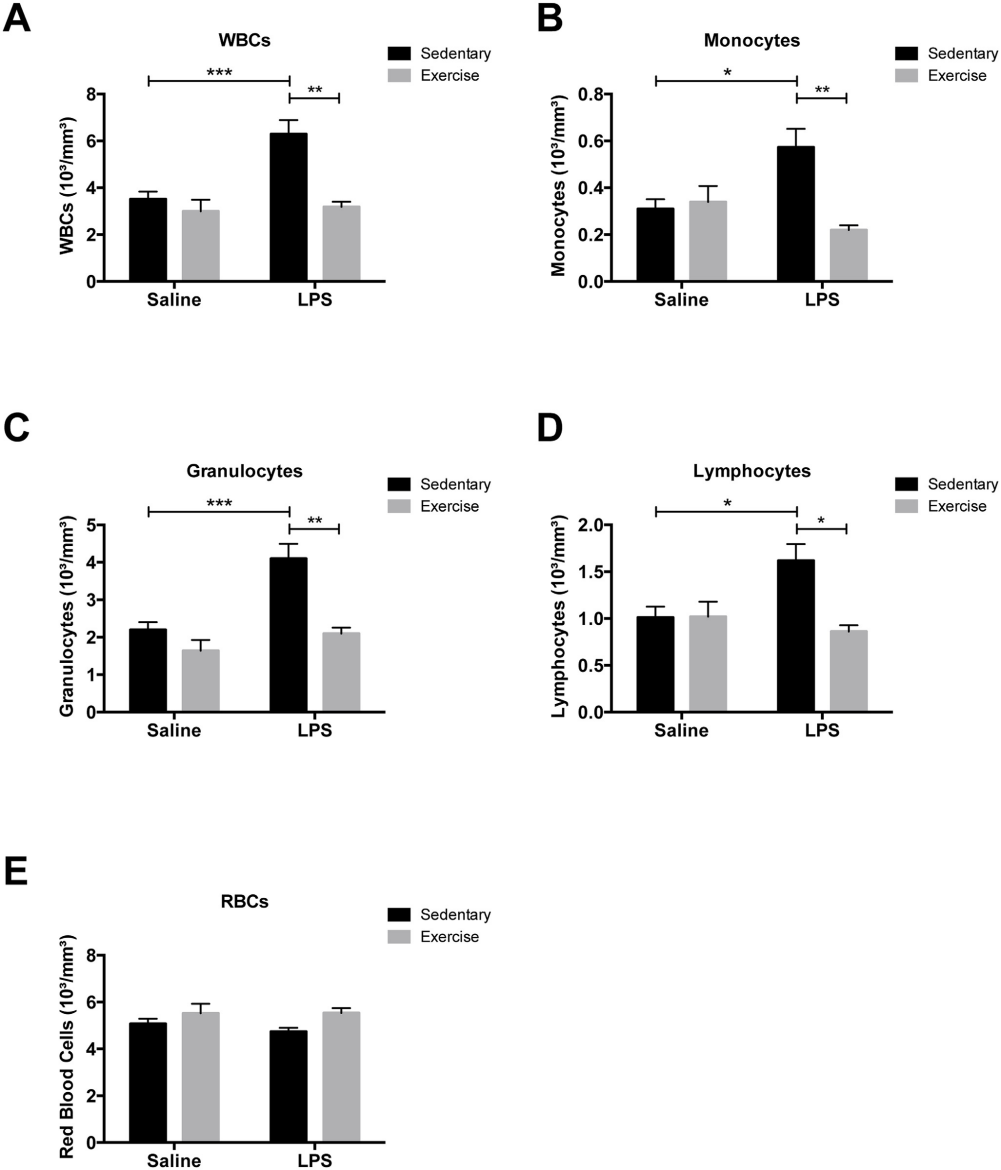
Exercise prevented LPS-induced increases in white blood cell counts. Exercise prevented LPS-induced increases in white blood cell (leukocyte) counts measured in blood samples collected on GD 17.5 (A). Exercise significantly abrogated LPS-induced increases in the number of circulating monocytes (B), granulocytes (C) and lymphocytes (D), but did not significantly alter the red blood cell count (E). Ex+LPS, n = 5; Ex+Saline, n = 5; Se+LPS, n = 11; Se+Saline, n = 10.

WBC counts measured from Ex+LPS-treated animals were significantly reduced compared with counts measured from Se+LPS-treated rats (Fig 1A; η^2^_Inflammation_ = 0.144; η^2^_Exercise_ = 0.217; η^2^_Interaction_ = 0.111); our previous study had revealed that WBC counts in this latter group of rats were significantly increased compared with sedentary control (saline-treated) rats [7]. Differential WBC analysis (Fig 1) also revealed that exercise significantly prevented LPS-induced increases in the number of circulating monocytes (Fig 1B; η^2^_Inflammation_ = 0.025; η^2^_Exercise_ = 0.127; η^2^_Interaction_ = 0.178), granulocytes (Fig 1C; η^2^_Inflammation_ = 0.201; η^2^_Exercise_ = 0.237; η^2^_Interaction_ = 0.075) and lymphocytes (Fig 1D; η^2^_Inflammation_ = 0.045; η^2^_Exercise_ = 0.126; η^2^_Interaction_ = 0.006). Exercise did not affect red blood cell counts in LPS-treated animals (p = 0.12; Fig 1E).

### Exercise Attenuated Inflammation-Induced FGR

**Fig 2:**
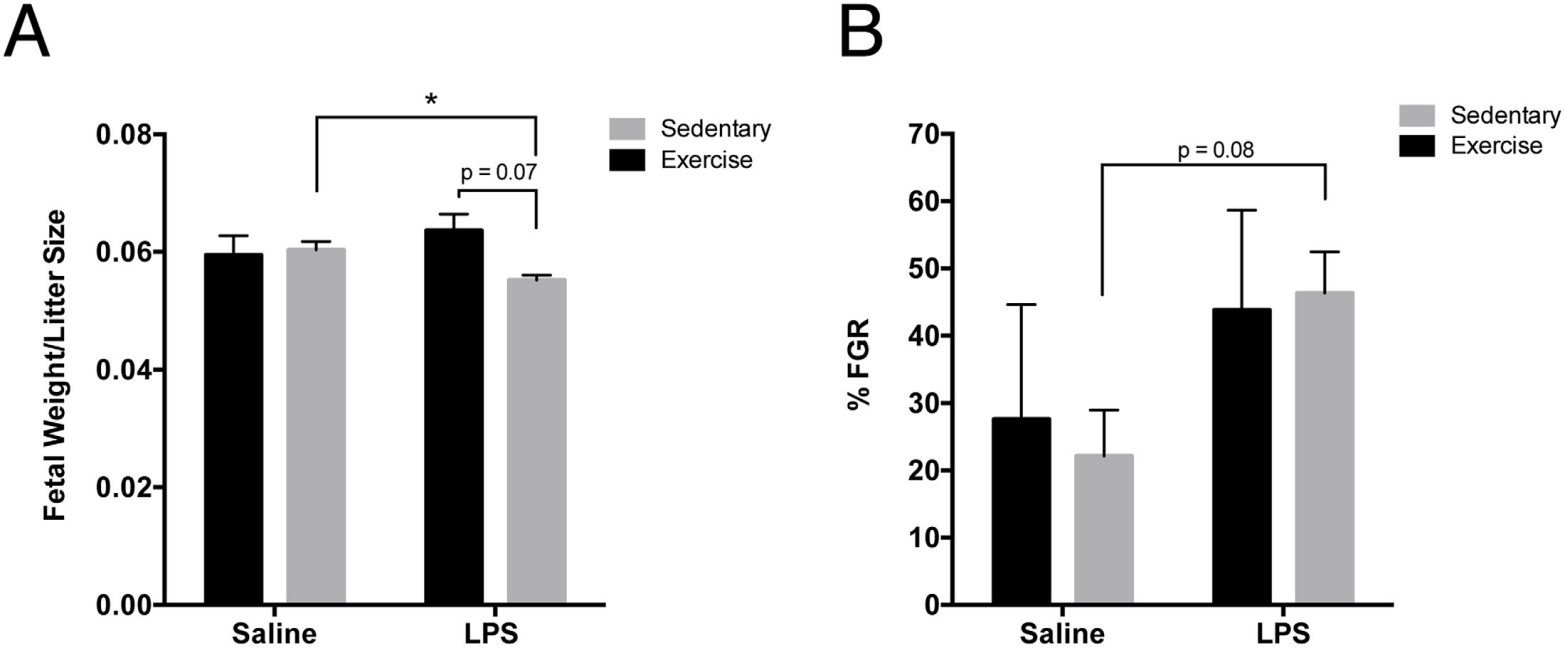
Exercise prevented LPS-induced fetal growth restriction. LPS significantly reduced fetal weights/litter size in sedentary rats (Se+LPS), whereas fetal weights in exercised rats treated with LPS (Ex+LPS) trended (p = 0.07) towards being significantly higher than in the Se+LPS cohort (A). Although the proportion of growth restricted fetuses trended towards being increased in Se+LPS rats than in Se+Saline rats [7], exercise did not significantly alter this proportion in the present study (B). FGR, fetal growth restriction. Ex+LPS (number of pups = 41, litter size varied 5 to 11), Ex+Saline (number of pups = 63; range in litter size = 4–19), Se+LPS (number of pups = 258), Se+Saline (number of pups = 305) (A); Ex+LPS (n = 5), Ex+Saline (n = 5), Se+LPS (n = 28), Se+Saline (n = 22) (B).

Our previously published data revealed that administration of LPS to sedentary pregnant rats induced FGR [7] (Fig 2A and 2B). This LPS-induced reduction in fetal weights observed in sedentary rats was not observed in exercised rats (Fig 2A). Moreover, weights of fetuses from Ex+LPS dams trended (p = 0.07) towards being significantly increased compared with weights of fetuses from Se+LPS dams (Fig 2A; η^2^_Inflammation_ < 0.001; η^2^_Exercise_ = 0.004; η^2^_Interaction_ = 0.006). Exercise did not significantly alter the of proportion of growth restricted fetuses (Fig 2B), and we observed no differences in total litter size (all implantation sites) or fetal viability (number of live pups in a litter) when all treatment groups were compared (data not shown).

### Exercise Normalized Coagulation Parameters Measured by TEG

**Fig 3:**
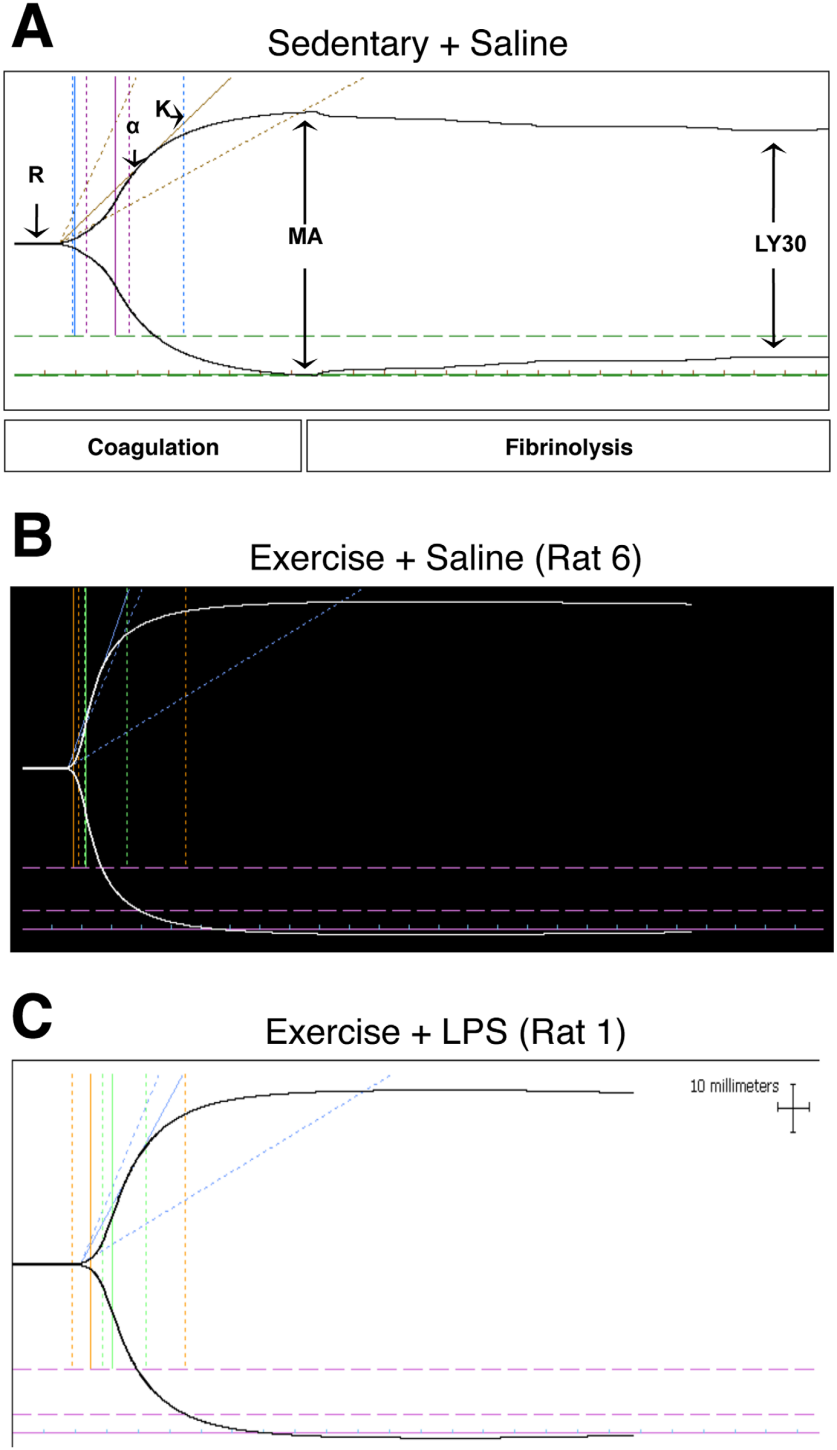
Exercise normalized thromboelastography parameters and tracings. Representative TEG trace revealing TEG parameters measured from blood collected from a Se+Saline control dam on GD 17.5 (A). TEG tracing from an Ex+Saline dam on GD 17.5 (B). TEG tracing from an Ex+LPS dam on GD 17.5 (C). R, time to clot formation; α, speed of clot propagation; K, rate of clot formation; MA, strength/stability of clot; CI, clotting index; LY30, percent clot dissolution in 30 min (%).

TEG coagulation parameters obtained from exercised rats (Saline and LPS) were compared with our previously published coagulation parameters obtained from sedentary rats (Saline and LPS) [19] (Table 1). Our data here reveal that exercise normalized coagulation parameters (Fig 3). Of the four rats in the Ex+Saline group, three had normal TEG parameters when compared with Se+Saline rats (Fig 3A and 3B). One rat (Rat 9) had TEG parameters indicative of a hypocoagulable state with more than two parameters falling beyond the reference ranges for normal pregnant rats, including increased K and decreased alpha angle and MA (Table 1).

**Table 1:** Thromboelastography parameters assessed on GD 17.5.

	R	K	α angle	MA	CI	LY30	Coagulopathy^#^
**Exercise + LPS** (n = 5)	10.78 (7.5–13.8)	3.68 (2.3–7.2)	50.86 (31.8–61.5)	62.7 (48.1–71.9)	1.54 (-1.1–3.1)	0.34 (0–1.7)	
Rat 1	10.4	2.8	53.7	71.9	3.1	0.0	Normal
Rat 2	9.9	2.6	56.6	59.0	1.0	0.0	Normal
Rat 3	13.8	7.2	31.8	48.1	-1.1	1.7	Hypocoagulable
Rat 4	12.3	3.5	50.7	71.4	2.6	0.0	Normal
Rat 5	7.5	2.3	61.5	63.1	2.1	0.0	Normal
**Exercise + Saline** (n = 4)	9.8 (8.6–10.9)	3.98 (2.1–8.1)	46.28 (29.2–62.9)	59.55 (52.8–71.8)	1.5 (0.8–3.3)	1.15 (0–2.6)	
Rat 6	8.6	2.1	62.9	71.8	3.3	0.0	Normal
Rat 7	10.9	2.8	36.6	56.1	0.8	2.6	Normal
Rat 8	10.8	4.4	42.1	57.5	0.9	2.0	Normal
Rat 9	8.9	8.1	29.2	52.8	1.0	0.0	Hypocoagulable
**Sedentary + LPS**^a^,[19] (n = 13)	8.9 (2.4–21.5)	2.5 (0.8–7.4)	60.6 (25.2–78.4)	67.4 (36.9–82.3)	3 (-3.3–5.8)	5.4 (0–47.5)	
**Sedentary + Saline**^a^,[19] (n = 9)	11.6 (7.9–18.2)	3.6 (1.8–5.9)	51.6 (33.2–65)	62.5 (56.7–71.5)	1.2 (-0.9–3.4)	5.1 (0–13.1)	

^a^Historical data from previoulsy published work [19]. All values presented as mean (minimum—maximum); R, time for clot formation; α, speed of clot propagation; K, rate of clot formation; MA, strength/Stability of clot; CI, clotting index; LY30, percent of clot dissolution in 30 min (%).

^#^Coagulopathy was compared with Sedentary + Saline group.

The TEG parameters assessed from blood collected from four of the five rats from the Ex+LPS group were not significantly different from TEG parameters assessed from blood samples collected from the Se+Saline group (Table 1; Fig 3A and 3C). For the remaining animal (Rat 3; Table 1), an increased K combined with reduced α angle, MA and CI was indicative of a hypocoagulable state. Overall, exercise normalized LPS-induced coagulation parameters as evaluated by TEG.

### Exercise Did Not Affect Maternal Weight Gain

Exercise did not affect maternal weight gain over the course of saline or LPS treatment when compared with sedentary rats. Two-way ANOVA comparing the change in maternal weight measured between GD 13.5 (onset of treatment) and GD 17.5 (endpoint) revealed that variations in maternal weight at endpoint are attributable to LPS treatment and not exercise (data not shown).

## Discussion

Here we describe the effect of moderate-intensity maternal exercise before and during pregnancy on preventing LPS-induced FGR and decreasing the intensity of the associated maternal inflammation and hemostatic alterations. The results of this study are in agreement with similar data reported in mouse studies in which voluntary exercise performed before and during pregnancy revealed positive effects on maternal outcomes. In those studies, voluntary maternal exercise was associated with better control of blood pressure during pregnancy, reduced proteinuria (albumin/creatinine ratio), decreased oxidative stress increasing placental antioxidant capacity, and reduced maternal and placental circulating sFlt-1 levels [35–37]. Despite the beneficial effects of exercise reported, voluntary exercise can result in intervention bias as the intensity of exercise experienced by each animal may differ. There is evidence that animals voluntarily reduce their physical activity later in gestation and that this reduction in activity varies between individuals [35, 36]. Though the reason for this variance is not well described, it is possible that physiological and biomechanical changes during pregnancy (*i*.*e*. weight gain and cardiovascular adaptations) play a role. In consideration of such inter-animal variability, we designed our experiments such that all rats were exposed to the same exercise regime throughout the study.

In our study, seven weeks of moderate-intensity exercise (including the pre-pregnancy training phase and exercise during gestation) prevented LPS-induced increases in white blood cell counts, normalized coagulation parameters and attenuated the development of LPS-induced coagulopathies. In addition, we observed a beneficial effect of maternal exercise on fetal outcomes such that exercise prevented LPS-induced FGR. Our data align with previous studies revealing that voluntary exercise ameliorates poor fetal growth [35, 36]. However, work from Rocha and colleagues revealed that maternal exercise (swimming) did not increase fetal weight in spontaneously hypertensive (SHR) rats [38]. In contrast to our model, in that study, rats began exercise (20 minutes of daily swimming initially; increased gradually to one hour per day until GD 20) on GD 7 and exercise promoted (rather than attenuated) FGR when compared with sedentary control rats [38]. Whereas these data suggest that exercise initiated during an established pregnancy may be detrimental as a result of increased maternal physiological demands and cardiovascular overload [36], our current study reveals that exercise initiated prior to pregnancy and consistently maintained throughout gestation may be beneficial.

While exercise promotes positive physiological adaptations during pregnancy [35, 36], forced exercise could also induce physiological stress [39]. We designed our study with a regimented protocol in order to control exercise intensity and duration. Moreover, we chose to model moderate-intensity exercise (65% of maximum exercise ability) because this level of exercise has been associated with maternal benefits and, importantly, because it has been recommended for pregnant women by the American College of Obstetrics and Gynecology [33, 40]. As our data reveal, though moderate-intensity exercise was advantageous, it did not completely prevent LPS-induced maternal and fetal alterations. Therefore, it is possible that exercise at a different level of intensity (*i*.*e*. high or low) may be a more effective option with broader outcomes. Indeed, there is evidence that differing exercise intensities during pregnancy have variable effects on maternal and neonatal health outcomes [41, 42].

To the best of our knowledge, this study provides the first evidence that moderate-intensity maternal exercise before and during pregnancy attenuates inflammation and its associated hemostatic alterations in a rat model. TEG is an effective tool for the evaluation of global hemostatic changes associated with both normal pregnancy [43] and adverse pregnancy outcomes [44]. Moreover, we previously demonstrated that inflammation-induced maternal hemostatic alterations, detected systemically using TEG, are comparable to hemostatic alterations detected locally at the utero-placental interface in a model of inflammation-induced fetal demise [18]. Though some studies have described a pro-thrombotic effect of high intensity exercise (>75% of VO_2max_) [45, 46], there is evidence that exercise prevents thrombosis during hospitalization and prolonged bedtime periods in non-pregnant individuals, and that exercise improves the hemostatic profile [47, 48]. In the present study, exercise normalized LPS-induced alterations in TEG parameters such that the values were not different from those of sedentary, saline-treated, control rats. The use of TEG has been an important tool for the evaluation of the coagulation index in rats [18, 19] and in this study TEG was used to assess individual hemodynamic changes in response to LPS and exercise. Moreover, our findings are consistent with previous publications that report a beneficial effect of exercise on coagulation parameters and an overall enhanced fibrinolytic state [47, 49].

The causative link between exaggerated maternal inflammation and development of pregnancy complications is becoming prominent [50] despite the fact that the initiating factors precipitating inflammation have yet to be conclusively identified. We chose to model inflammation-induced pregnancy complications using systemic LPS exposure in pregnant rats. Our model is based, in part, on studies by Faas and colleagues who first described the use of low-dose LPS infusion to model a PE-like syndrome in pregnant rats [51, 52]. Though infection with LPS-positive bacteria is relatively uncommon in pregnant women, it is well established that infection is associated with the onset of pre-term labour in approximately 40% of cases [53]. Moreover, there is evidence that periodontal disease, in which bacterial LPS is the most prominent pro-inflammatory factor, is associated with miscarriage [54], low birthweight [55] and PE [56] as assessed using both human observational data and *in vivo* animal models [57].

Exercise is known to reduce the release of pro-inflammatory cytokines, stimulate the release of anti-inflammatory cytokines and diminish the physiological consequences of exaggerated inflammation. In light of accumulating evidence for a role of abnormal inflammation in the development of pregnancy complications [50], including FGR [7] and spontaneous pregnancy loss [18, 19, 23], the results of our study warrant further investigation of the potential benefits of exercise for women with clearly identified complications (*e*.*g*. recurrent pregnancy loss) associated with aberrant inflammation.
